# atlasBREX: Automated template-derived brain extraction in animal MRI

**DOI:** 10.1038/s41598-019-48489-3

**Published:** 2019-08-21

**Authors:** Johannes Lohmeier, Takaaki Kaneko, Bernd Hamm, Marcus R. Makowski, Hideyuki Okano

**Affiliations:** 1 Charité Universitätsmedizin Berlin, Radiology, Berlin Germany; 2 Center for Brain Science Institute, RIKEN, Marmoset Neural Architecture, Wako-shi, Saitama Japan; 3 Department of Physiology, Keio University School of Medicine, Tokyo, Japan

**Keywords:** Image processing, Computational neuroscience, Magnetic resonance imaging

## Abstract

We proposed a generic template-derived approach for (semi-) automated brain extraction in animal MRI studies and evaluated our implementation with different animal models (macaque, marmoset, rodent) and MRI protocols (T1, T2). While conventional MR-neuroimaging studies perform brain extraction as an initial step priming subsequent image-registration from subject to template, our proposed approach propagates an anatomical template to (whole-head) individual subjects in reverse order, which is challenging due to the surrounding extracranial tissue, greater differences in contrast pattern and larger areas with field inhomogeneity. As a novel approach, the herein introduced brain extraction algorithm derives whole-brain segmentation using rigid and non-rigid deformation based on unbiased anatomical atlas building with a priori estimates from study-cohort and an initial approximate brain extraction. We evaluated our proposed method in comparison to several other technical approaches including “Marker based watershed scalper”, “Brain-Extraction-Tool”, “3dSkullStrip”, “Primatologist-Toolbox”, “Rapid Automatic Tissue Segmentation” and “Robust automatic rodent brain extraction using 3D pulse-coupled neural networks” with manual skull-stripping as reference standard. ABX demonstrated best performance with accurate (≥92%) and consistent results throughout datasets and across species, age and MRI protocols. ABX was made available to the public with documentation, templates and sample material (https://www.github.com/jlohmeier/atlasBREX).

## Introduction

**Figure 1:**
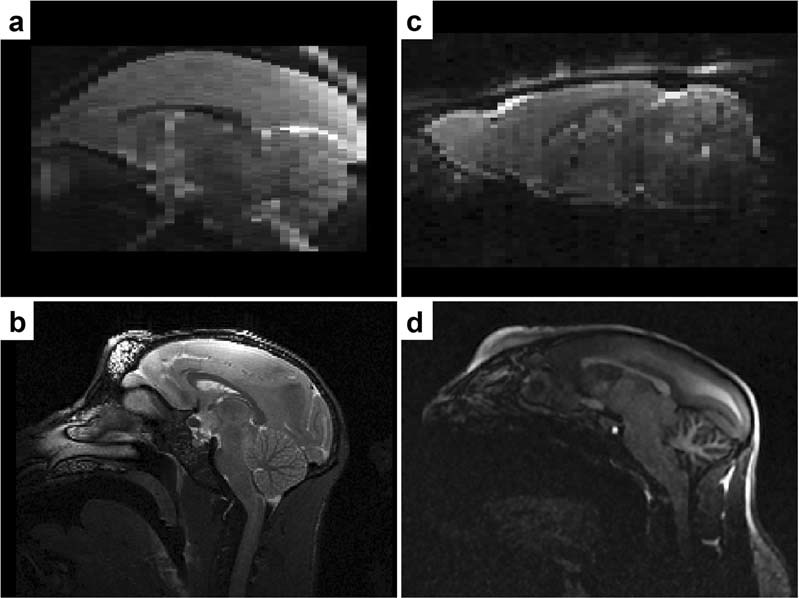
Challenges in skull-stripping animal MR-neuroimaging. Illustration of common difficulties when skull-stripping animal MRI (top-left: marmoset [9.4 T Biospec, Bruker, Germany]; top-right: rat [9.4 T Biospec, Bruker, Germany]; bottom-left: rhesus macaque [3 T Prisma, Siemens, Germany]; bottom-right: marmoset [3 T Prisma, Siemens, Germany]), such as low image resolution (**a**,**c**), strong field inhomogeneity (**a**–**d**) or greater field-of-view (**b,d**) with larger areas of non-brain tissue.

Brain extraction, also referred as skull-stripping or whole-brain segmentation, describes the process of extracting the brain from the surrounding extracranial tissue. In MRI studies, it is common that this procedure is implemented at an early stage, as it plays an important role for further processing, such as spatial normalisation, surface reconstruction and structural analysis^1^. Manual delineation is considered technical standard, but it demands high time investment, experience and neuroanatomical knowledge. Hence, there is need for automated technical alternatives, which are less operator-dependent. Several (semi-) automated (hybrid) approaches were developed for human neuroimaging thus far^1^, but present a high degree of specialisation due to a priori estimates. Therefore, established technical approaches for human neuroimaging are often not compatible with animal MRI and the adaption can be demanding due to interspecies differences in brain size, shape and tissue contrast as well as differences in MRI scanners, magnetic field strengths, radiofrequency coils and MRI protocols. A common challenge in skull-stripping animal MR-neuroimaging is the presence of more severe field inhomogeneity, which is attributable to non-uniformity in radiofrequency coils. As illustrated in Fig. 1, both pattern of occurrence (see heterogeneous gradient) as well as the severity of distortion are subject to variation, which affects the performance of processing algorithms that infer information from image intensity. Further challenges arise from low-resolution images (see Fig. 1a,c), such as in functional and diffusion MRI studies.

In recent years, neuroimaging studies with animal models, such as macaque^2^, marmoset^3,4^ and rodent^5^, gained in significance due to their contributions to understanding the central nervous system^6,7^. To date, however, there are only few technical approaches available that can be applied to animal MRI, such as Marker based watershed scalper (MBWSS)^8^, Brain-Extraction-Tool (BET)^9^, 3dSkullStrip (3DSS) as part of the Analysis of Functional NeuroImages (AFNI) package^10^, Primatologist-Toolbox (PRIMA)^11^, Rapid Automatic Tissue Segmentation (RATS)^12^ and Robust automatic rodent brain extraction using 3D pulse-coupled neural networks (PCNN3D)^13^. However, it is common that results are below standard and require further manual intervention. Hence, there is demand for more robust brain extraction algorithms in animal MRI.

**Figure 2:**
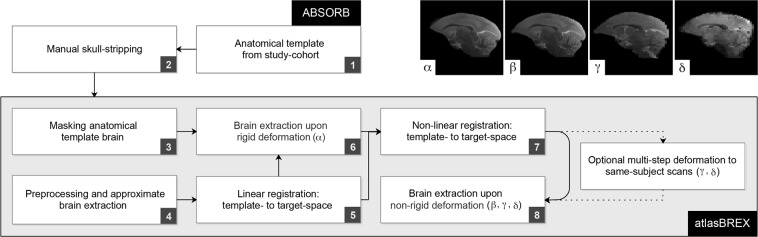
Workflow of atlasBREX for (semi-) automated brain extraction. In a first step (1), an unbiased anatomical template is constructed from the respective study-cohort using ABSORB, which is then subject to manual (hybrid) skull-stripping (2). Upon preprocessing in order to optimize the following image registration and initial approximate brain extraction (3), rigid and non-rigid deformation (5 and 7) fields are computed from template- to target-space, which are applied to the template mask in order to compute a subject-specific brain mask (6 and 8). Optional multi-step deformation to other target volumes, such as structural low-resolution (γ) and functional scans (δ), can be performed. Panels on the top-right demonstrate results from an adult marmoset upon rigid (α) and non-rigid (β) deformation as well as the results from the corresponding structural low-resolution (γ) and functional scans (δ).

Therefore, we proposed a generic template-derived approach for animal neuroimaging: We present atlasBREX (ABX), a semi-automated processing pipeline that propagates skull-stripping of an anatomical template built from the study-cohort after rigid and non-rigid deformation to each individual subject (see Fig. 2). First, in a practical and unbiased manner, an anatomical study-specific template is computed from all individual subjects (see Fig. 2, step 1) using an iterative hierarchical group-wise registration framework, Atlas Building by Self-Organized Registration and Bundling (ABSORB)^14^. Next, the study-specific anatomical template is subject to manual (hybrid) skull-stripping (see Fig. 2, step 2). In the following steps, rigid and non-rigid deformation fields are computed from template- to target-space (see Fig. 2, step 5 and 7), which are applied to the template mask in order to compute a subject-specific brain mask (see Fig. 2, step 6 and 8).

While spatial transformation is conventionally performed after image preprocessing (including brain extraction) with the objective to align images within or across individuals, our proposed approach is based on the backpropagation of an anatomical template to the (whole-head) individual subject, which is challenging due to the surrounding extracranial tissue, greater differences in contrast pattern and larger areas with strong field inhomogeneity (see Fig. 1).

Therefore, as a novel approach, the herein introduced brain extraction algorithm derives whole-brain segmentation using rigid and non-rigid deformation based on unbiased anatomical atlas building with a priori estimates from the study-cohort and an initial approximate brain extraction. Thereby, our proposed approach achieves accurate and consistent results for an entire study population using only a single template, which exhibits high anatomical conformance and similarity in contrast. On the contrary, available templates, which were not built from the respective study population, can negatively impact the performance of registration algorithms due to greater divergence in brain morphology and differences in contrast pattern, originating from MRI scanner, radiofrequency coil or imaging protocol. In order to address some of the challenges described above, preprocessing operations, such as voxel-scaling, bias-field correction and intensity normalisation, were additionally implemented into the processing stream.

We evaluated ABX across different MRI protocols (T1, T2) with macaques, marmosets and rodents and compared its performance to several other technical methods.

## Method

### Datasets and MR-imaging protocols

Rhesus macaque (n = 32, age 0.5–36 months) datasets from the open, longitudinal UNC-Wisconsin Neurodevelopment Rhesus (UNCW-NRh) database^15^ were obtained featuring preprocessed (AutoSeg2, N4BiasFieldCorrection and BRAINSfit3 registration to Emory-UNC atlas) T1-/T2-weighted scans (Waisman Lab, University of Wisconsin Madison, 3 T MRI, GE Medical, Milwaukee WI; T1W/GE-BRAVO, TR/TE = 8.684/3.652 ms, FOV = 140 × 140 mm, matrix = 256 × 256, thickness = 0.8 mm, resolution = 0.55 × 0.55 × 0.8 mm; T2W/3D-FSECUBE, TR/TE = 2500/87 ms, FOV = 154 × 154 mm, matrix = 256 × 256, thickness = 0.6 mm, resolution = 0.6 × 0.6 × 0.6 mm) including results from manual hybrid brain extraction using Atlas-Based-Classification and manual intervention, which were used as reference standard.

All procedures were approved by Wako Animal Experiment Committee (Animal Care and Use Committee), RIKEN, and all experiments were performed in accordance with the approved guidelines and regulations. The following datasets were obtained:

Adult marmoset acquired at RIKEN Brain Science Institute (9.4 T MRI, Bruker, Germany; T2W/RARE, TR/TE = 8000/32 ms, FOV = 48 × 38.5 mm, matrix = 320 × 256, thickness = 0.3 mm, slices = 97, resolution = 0.15 × 0.15 × 0.3 mm, RARE-factor = 8; T2*W/Gradient-EPI, TR/TE = 2000/16 ms, FOV = 42 × 22 mm, matrix = 84 × 44, thickness = 1 mm, slices = 35, resolution = 0.5 × 0.5 × 1 mm). At Central Institute for Experimental Animals (CIEA) juvenile (n = 6, age 6–12 months) marmoset (7 T MRI, Bruker, Germany; T1W/MPRAGE, TR/TE = 14/45 ms, FOV = 50 × 50 mm, matrix = 192 × 192, thickness = 0.27 mm, slice = 120, resolution = 0.26 × 0.26 × 0.27 mm), adult (n = 5) marmoset (3 T MRI, Siemens, Germany; T2W, TR/TE = 3200/562 ms, FOV = 128 × 128 mm, matrix = 256 × 256, thickness = 0.5 mm, slice = 224, resolution = 0.5 × 0.5 × 0.5 mm; one sample excluded for quality reasons) and adult (n = 6) mouse (7 T MRI, Bruker, Germany; T2W/RARE, TR/TE = 6100/48 ms, FOV = 1.92 × 1.92 mm, matrix = 256 × 256, thickness = 0.3 mm, slice = 52, resolution = 0.075 × 0.075 × 0.3 mm, RARE-factor = 8) were obtained. Reference images were skull-stripped using a manual hybrid approach with BrainSuite (v16a1)^16^.

### Building templates from the study-cohort using ABSORB

A hierarchical group-wise registration framework, Atlas Building by Self-Organized Registration and Bundling (ABSORB)^14^, was used for unbiased template building (with histogram-matching and affine-registration; neighbourhood-size: 3; smoothing-kernel: 3,2,1; maximum-levels: 5; registration-to-mean: 3) built from the respective study-cohort (with whole-head images). Datasets were bias-field corrected (N4BiasFieldCorrection, ANTs) and aligned (3dAllineate, AFNI), where the built-in registration algorithm led to insufficient results. Gaussian convolution (3dmerge, AFNI) (smoothing-kernel: 0.3–0.5) was applied to templates after manual (hybrid) skull-stripping with BrainSuite (v16a1).

### atlasBREX with unbiased anatomical templates

atlasBREX (ABX) is a semi-automated processing pipeline that propagates skull-stripping of an anatomical template built from the study-cohort upon rigid and non-rigid deformation to each individual subject. It requires functional set-up of FMRIB’s Software Library (FSL, v5.0). Several optional features are available making use of Analysis of Functional NeuroImages (AFNI, v17.1.01) and Advanced Normalization Tools (ANTs, v2.1.0)^17^.

At first, a template mask is aligned to a subject using linear registration (FLIRT, FSL) between the template brain and an initial approximate brain extraction from Brain-Extraction-Tool v2 (BET, FSL), as shown in Fig. 2. Prior to the provisional brain extraction, voxel dimensions are adjusted to resemble the human brain and (optional) preprocessing can be applied, such as intensity normalisation (3dUnifize, AFNI), recommended for T1-weighted images, and bias-field correction (N4BiasFieldCorrection, ANTs), which compensates for field inhomogeneity. Changes during preprocessing are implemented on intermediates and, therefore, are not present in final results.

In the following steps, a subject-specific brain mask is computed from the non-rigid deformation field based on non-linear registration (FNIRT, FSL), which relies on the affine transformation matrix from the previous linear registration and a (dilated) reference mask based on the result from rigid deformation, which prevents adverse impact of surrounding extracranial tissue at full resolution during registration. Where additional scans from the same subject are available, such as low-resolution structural or functional scans, rigid and non-rigid transformation matrices can be applied upon an additional transformation step.

Prior to automated processing of an entire dataset, a brief interactive user-guided pilot run needs to be performed to determine a suitable intensity threshold (0.3, 0.5 or 0.8) for the initial approximate brain extraction.

### Evaluation of atlasBREX

For quantitative evaluation, image datasets from infant (n = 8, 0.5–1 months, T1/T2) and juvenile (n = 8, 24–36 months, T1/T2) rhesus macaques, juvenile marmosets (n = 6, 6–12 months, T1), adult marmosets (n = 5, T2) and adult mice (n = 6, T2) were used. ABX was utilized on each dataset (with a single set of parameters upon a brief pilot run to determine an intensity threshold) with an anatomical template built from the respective study-cohort. In addition to Brain-Extraction-Tool v2 (BET), several dedicated technical methods (3dSkullStrip (3DSS), Primatologist-Toolbox (PRIMA) and Marker based watershed scalper (MBWSS) dedicated to non-human primates; Robust automatic rodent brain extraction using 3-D pulse-coupled neural networks (PCNN3D), Rapid Automatic Tissue Segmentation (RATS) and 3dSkullStrip (3DSS) dedicated to rodents) were applied to each dataset. Brain-Extraction-Tool v2 (BET) was assessed with different fractional intensity thresholds (5 variants, thresholds from 0.1–0.9). 3dSkullStrip (3DSS) was evaluated with dedicated presets (“monkey”, “rat” and “marmoset”). Marker based watershed scalper (MBWSS) features an optimized version for macaques, which was evaluated with different parameters (one variant with default parameters with (built-in) bias-field correction; 2 variants with settings recommended by the author: bias-field correction, increased smoothing, mask refinement, increased radius of opening). Primatologist-Toolbox (PRIMA) was assessed using respective options for T1- and T2-weighting and presets for “macaque” and “mouse”. For MBWSS and BET, where more than one variant was available, the best result was chosen according to the highest Jaccard-Index. All datasets were analysed on the same multi-core hardware platform (32 × 2.70 Ghz Intel-CPU, 260 GB RAM) running Linux 3.10 Centos 64-bit with FMRIB’s Software Library (FSL v5.0.10), Analysis of Functional NeuroImages (AFNI v17.2.09) and Advanced Normalization Tools (ANTs v2.1.0). ABX typically required few hours for computation.

### Statistical analysis

After visual inspection, quantitative evaluation was performed with manual (hybrid) skull-stripping as reference standard and Jaccard-Index (1) as similarity metric (metrics reported show Jaccard-Index). Quantitative parameters from the respective groups were analysed for statistically significant difference compared to ABX (each group tested against the results from ABX) using Friedman test followed by Dunn’s post-hoc test. Adjusted p-value < 0.05 was considered statistically significant.

1 $$J({\rm{A}},\,{\rm{B}})=\frac{|A{\cap }^{}B|}{|A{\cup }^{}B|}$$

## Results

### Evaluation with infant and juvenile rhesus macaques

We evaluated our approach with infant and juvenile non-human primates, which  can be considered challenging due to differences in brain volume and shape with regard to fixed a priori estimates in most brain extraction algorithms. In an overall view, ABX (Mdn[IQR] = 0.95[0.03]) demonstrated best performance regardless of image contrast (T1 vs. T2) and developmental stage (infant vs. juvenile) outperforming BET (Mdn[IQR] = 0.87[0.06], p < 0.001), 3DSS (Mdn[IQR] = 0.86[0.24], p = 0.001), MBWSS (Mdn[IQR] = 0.72[0.18], p < 0.001) and PRIMA (Mdn[IQR] = 0.65[0.36], p < 0.001), as shown in Table 1.

**Table 1:** Results from comparative analysis.

	Friedman test	Dunn’s test	Mean-rank difference	Adjusted p-value
Infant rhesus macaque T1-weighted, n = 8	F = 16.60 p = 0.002	BET	18.00	p = 0.02 (*)
		3DSS	9.00	p = 0.62
		PRIMA	24.00	p < 0.001 (***)
		MBWSS	14.00	p = 0.11
Infant rhesus macaque T2-weighted, n = 8	F = 30.50 p < 0.001	BET	21.00	p = 0.004 (**)
		3DSS	2.00	p > 0.99
		PRIMA	13.00	p = 0.16
		MBWSS	29.00	p < 0.001 (***)
Juvenile rhesus macaque T1-weighted, n = 8	F = 25.90 p < 0.001	BET	18.00	p = 0.02 (*)
		3DSS	15.00	p = 0.07
		PRIMA	32.00	p < 0.001 (***)
		MBWSS	15.00	p = 0.07
Juvenile rhesus macaque T2-weighted, n = 8	F = 22.10 p < 0.001	BET	10.00	p = 0.46
		3DSS	20.00	p = 0.006 (**)
		PRIMA	−1.00	p > 0.99
		MBWSS	21.00	p = 0.004 (**)
Infant and juvenile rhesus macaque T1-/T2-weighted, n = 32	F = 49.38 p < 0.001	BET	67.00	p < 0.001 (***)
		3DSS	46.00	p = 0.001 (**)
		PRIMA	68.00	p < 0.001 (***)
		MBWSS	79.00	p < 0.001 (***)
Juvenile marmoset T1-weighted, n = 6	F = 17.73 p = 0.001	BET	16.00	p = 0.01 (*)
		3DSS	8.00	p = 0.58
		PRIMA	15.00	p = 0.02 (*)
		MBWSS	21.00	p < 0.001 (***)
Adult marmoset T2-weighted, n = 5	F = 15.52 p = 0.004	BET	15.00	p = 0.01 (*)
		3DSS	9.00	p = 0.29
		PRIMA	8.00	p = 0.44
		MBWSS	18.00	p = 0.001 (**)
Juvenile and adult marmoset T1-/T2-weighted, n = 11	F = 32.00 p < 0.001	BET	31.00	p < 0.001 (***)
		3DSS	17.00	p = 0.09
		PRIMA	23.00	p = 0.008 (**)
		MBWSS	39.00	p < 0.001 (***)
Adult mouse T2-weighted, n = 6	F = 21.73 p < 0.001	BET	19.00	p = 0.002 (**)
		3DSS	10.00	p = 0.27
		PCNN3D	2.00	p > 0.99
		RATS	19.00	p = 0.002 (**)

Comparative analysis between atlasBREX (ABX) and alternate technical methods (each group tested against the results from ABX) was performed using Jaccard-Index and Friedman test with Dunn’s post-hoc test. (*) p-value < 0.05, (**) p-value < 0.01, (***) p-value < 0.001.

**Figure 3:**
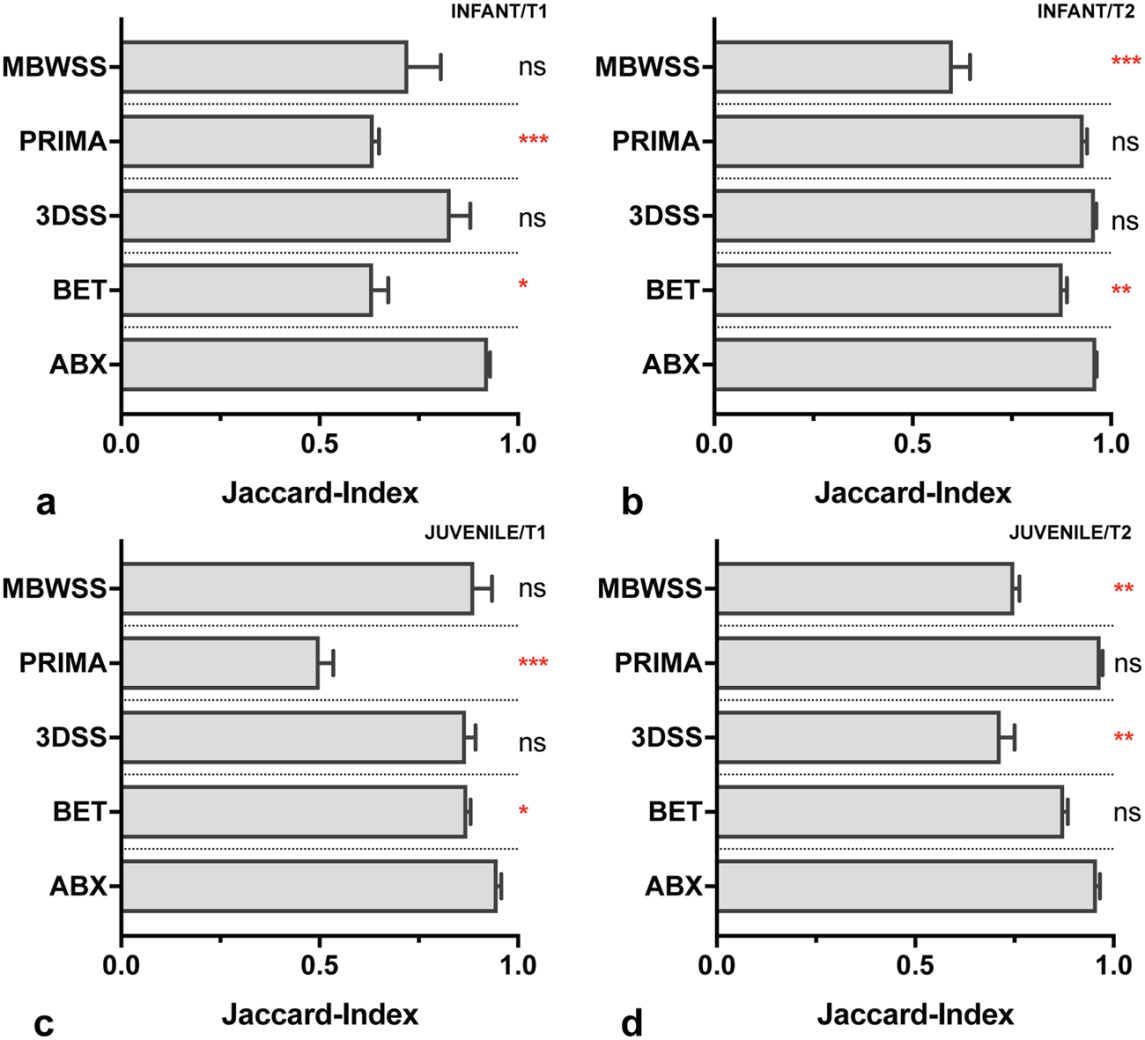
Evaluation with infant and juvenile rhesus macaques. Bar graph diagrams (**a**–**d**) demonstrating results from atlasBREX (ABX), Brain-Extraction-Tool v2 (BET), 3dSkullStrip (3DSS), Primatologist-Toolbox (PRIMA) and Marker based watershed scalper (MBWSS) for each dataset (4 groups, each n = 8). Results from alternate technical methods were compared to ABX using Friedman test followed by Dunn’s post-hoc test. (*) p-value < 0.05, (**) p-value < 0.01, (***) p-value < 0.001, (ns) non-significant. Mdn, IQR.

For infant macaque datasets, as shown in Fig. 3a,b, 3DSS (T1, T2) and PRIMA (T2) achieved decent accuracy, however, visual inspection revealed that laborious manual intervention was inevitable for results from 3DSS (T1), while minor adjustments were needed for results from PRIMA (T2). Regarding juvenile macaque datasets (see Fig. 3c,d), PRIMA (T2) showed reliable performance. Although no statistically significant differences were apparent for MBWSS (T1), 3DSS (T1) and BET (T2), visual inspection showed that extensive manual corrections were still required. PRIMA presented better performance with T2-weighted datasets, while MBWSS showed higher accuracy for datasets with T1-weighting. Contrarily, ABX demonstrated robust and consistent brain extraction throughout datasets. Inaccurate delineation from ABX was apparent in a single case (Jaccard-Index = 0.76), which would have required parameter optimisation.

**Figure 4:**
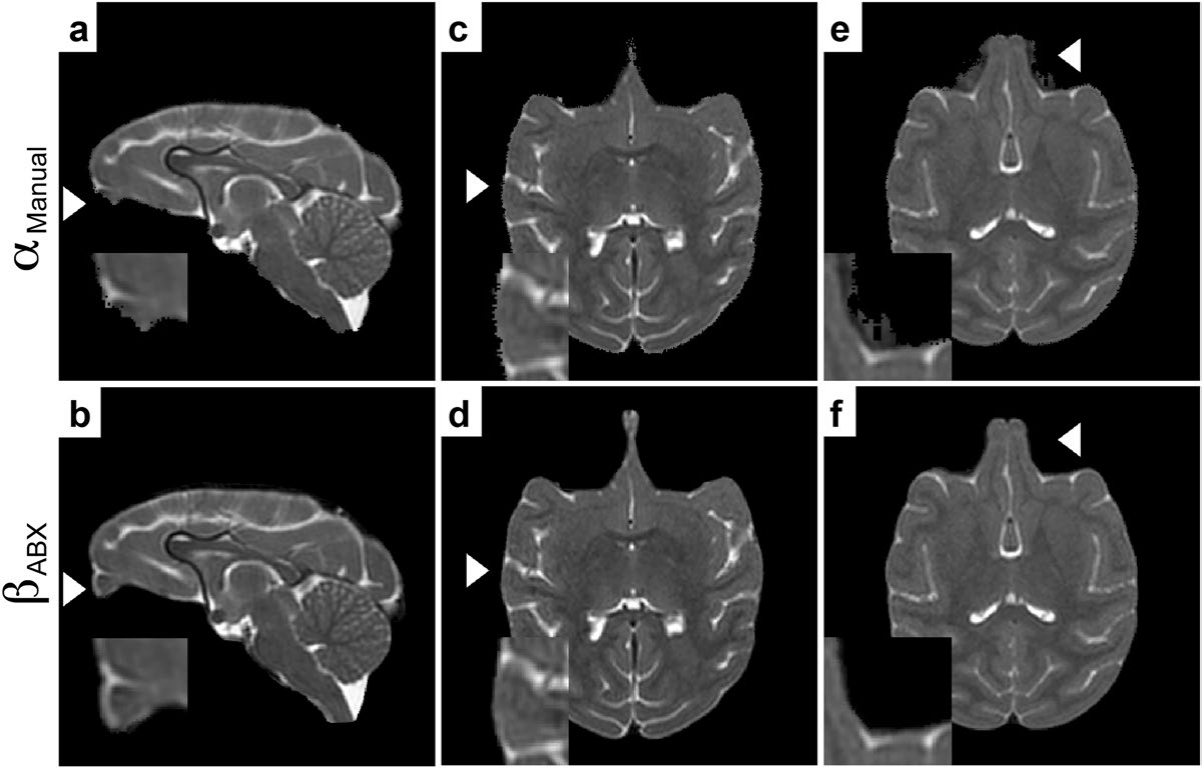
Comparison between atlasBREX (ABX) and manual delineation from UNC-Wisconsin Rhesus Macaque Neurodevelopment Database. Representative cases from juvenile rhesus macaque (n = 8, T2) presenting common disadvantages of manual (hybrid) brain extraction (α), which is operator-dependent and time-consuming, compared to (semi-) automated skull-stripping (β) using ABX. First two panels (**a**,**b**) show common erosion of the olfactory bulb. Jagged edges with marginal erosion of brain tissue are depicted in the following two panels (**c**,**d**). Last panels (**e**,**f**) demonstrate inaccurate delineation. White arrow indicates the magnified region-of-interest.

Manual definition of the segmentation boundary is tedious and highly operator-dependent, which makes it prone to human error, e.g. unintentional erosion of brain voxels (see Fig. 4a), remaining non-brain tissue (see Fig. 4e) or insufficient masking with jagged edges (see Fig. 4c). In contrast, as shown in Fig. 4b,d,f, results from ABX presented consistent and accurate brain extraction with a high degree of standardisation through automated processing using a single set of parameters.

### Evaluation with juvenile and adult marmosets

We evaluated ABX with datasets from juvenile and adult marmosets obtained with different magnetic field strengths (3 T vs. 7 T) and MRI protocols (T1 vs. T2). In particular, the 3 T (adult) marmoset dataset, acquired on a device originally designed for human imaging, is technically demanding due to a greater field-of-view with larger differences in contrast pattern. ABX (Mdn[IQR] = 0.95[0.02]) presented consistent and highest accuracy across datasets (see Table 1), which was followed by 3DSS (Mdn[IQR] = 0.85[0.08], p = 0.09), PRIMA (Mdn[IQR] = 0.82[0.18], p = 0.008), BET (Mdn[IQR] = 0.77[0.08], p < 0.001) and MBWSS (Mdn[IQR] = 0.34[0.40], p < 0.001).

**Figure 5:**
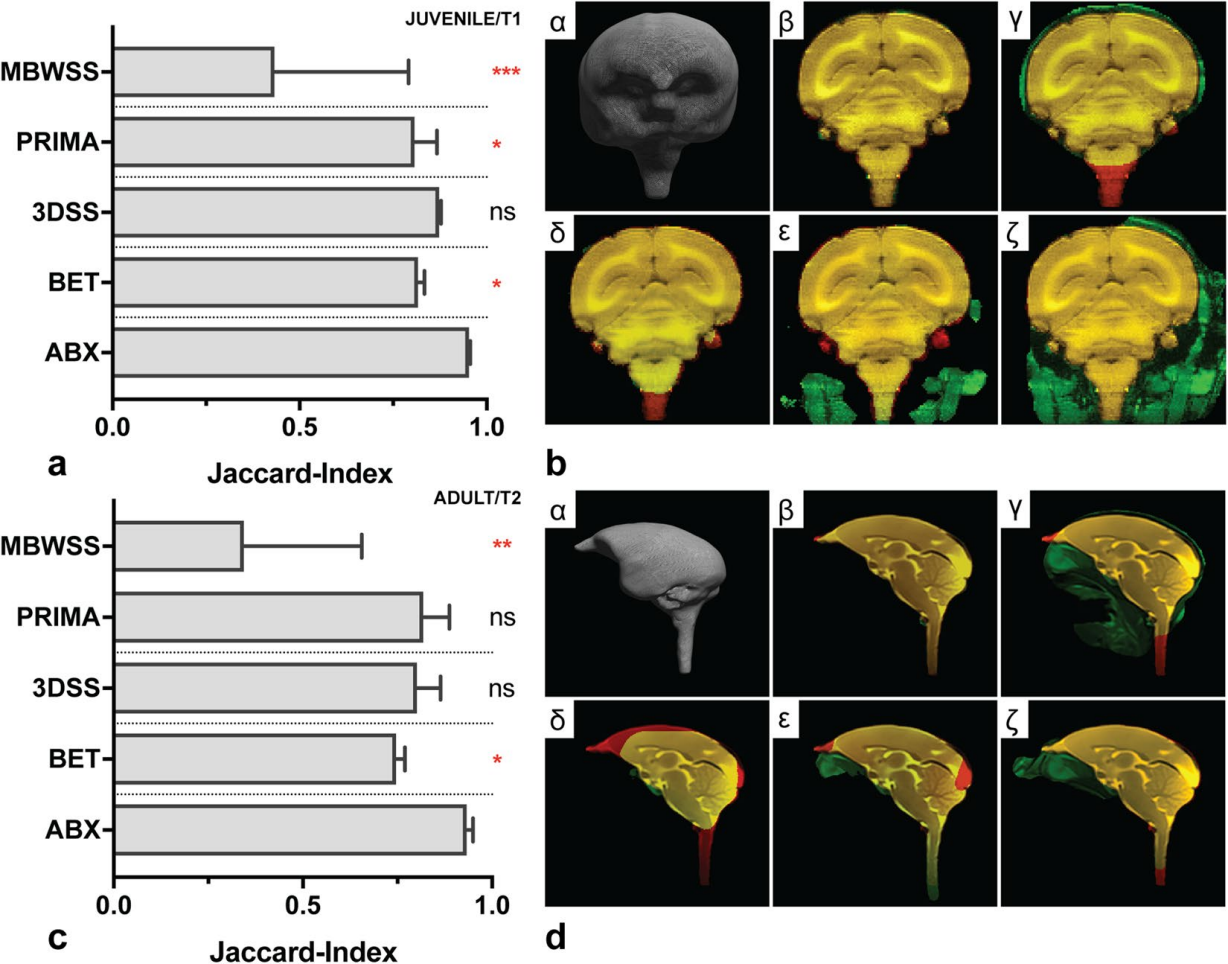
Evaluation with juvenile (n = 6, T1) and adult (n = 5, T2) marmosets. Bar graph diagrams (**a**,**c**) showing results from atlasBREX (ABX), Brain-Extraction-Tool v2 (BET), 3dSkullStrip (3DSS), Primatologist-Toolbox (PRIMA) and Marker based watershed scalper (MBWSS). Results from alternate technical methods were compared to ABX using Friedman test followed by Dunn’s post-hoc test. (*) p-value < 0.05, (**) p-value < 0.01, (***) p-value < 0.001, (ns) non-significant. Mdn, IQR. For juvenile (**b**) and adult (**d**) marmoset, results from each method are demonstrated for a representative subject in coronal plane. First image (α) shows a 3D surface model computed from the result of ABX. The following images (β, γ, δ, ε, ζ) demonstrate the same slice using a three-coloured overlay (yellow colour indicates voxel match, while red (brain tissue erosion) or green (residual non-brain tissue) colour show mismatch) between reference and the respective result from ABX (β), BET (γ), 3DSS (δ), PRIMA (ε) and MBWSS (ζ).

Although there were no statistically significant differences for 3DSS (for both datasets) and PRIMA (for the adult marmoset dataset), visual inspection showed that (extensive) manual intervention was still required (see Fig. 5b,d). Brain voxel erosion (see red colour), notably affected the rostral/anterior and caudal/posterior brain regions (see Fig. 5,d, panel δ (3DSS) and ε (PRIMA)). Moreover, large areas with non-brain tissue (see green colour) remained, demonstrated in Fig. 5b,d, respectively panel ε (PRIMA). In contrast, ABX presented accurate segmentation of the marmoset brain, illustrated with brain models in Fig. 5b,d, respectively panel α, particularly with regard to the olfactory bulb.

### Evaluation with adult mice

**Figure 6:**
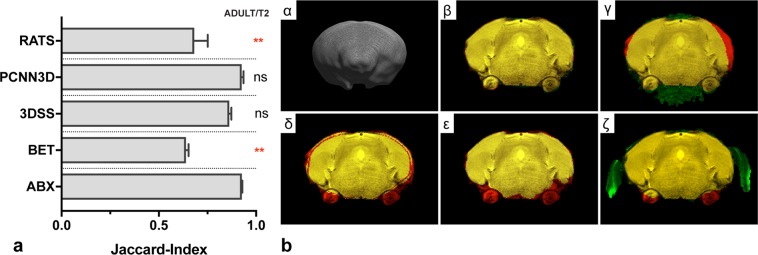
Evaluation with adult (n = 6, T2) mice. First panel (a) presents a bar graph diagram from results of atlasBREX (ABX), Brain-Extraction-Tool v2 (BET), 3dSkullStrip (3DSS), Robust automatic rodent brain extraction using 3-D pulse-coupled neural networks (PCNN3D) and Rapid Automatic Tissue Segmentation (RATS). Results from alternate technical approaches were compared to ABX using Friedman test followed by Dunn’s post-hoc test. (**) p-value < 0.01, (ns) non-significant. Mdn, IQR. Second panel (b) demonstrates results from each method for a representative subject in coronal plane. First image (α) shows a 3D surface model computed from the result of ABX. The following images (β, γ, δ, ε, ζ) demonstrate the same coronal slice using a three-coloured overlay (yellow colour indicates voxel match, while red (brain tissue erosion) or green (residual non-brain tissue) colour show mismatch) between reference and the respective result from ABX (β), BET (γ), 3DSS (δ), PCNN3D (ε) and RATS (ζ).

Moreover, we evaluated ABX with a rodent dataset, which is challenging to most brain extraction algorithms considering the vastly different brain morphology and difference in contrast between brain and scalp when compared to the primate brain. As shown in Fig. 6, ABX (Mdn[IQR] = 0.93[0.01]) demonstrated accurate results similar to its performance shown with non-human primates. While PCNN3D (Mdn[IQR] = 0.93[0.02], p > 0.99) and 3DSS (Mdn[IQR] = 0.86[0.02], p = 0.27) achieved similar performance (see Fig. 6a), visual inspection suggested (minor) manual corrections in individual cases. Both RATS (Mdn[IQR] = 0.68[0.16], p = 0.002) and BET (Mdn[IQR] = 0.64[0.02], p = 0.002) provided insufficient results, as demonstrated in Fig. 6b, panel γ (BET) and ζ (RATS).

## Discussion

We introduced atlasBREX (ABX), a generic automated template-derived approach for brain extraction, and evaluated it with different animal models (macaque, marmoset and rodent) and MRI protocols (T1, T2). ABX demonstrated best performance and presented accurate and consistent results throughout datasets. We showed that (semi-) automated brain extraction using ABX can achieve results similar to manual (hybrid) skull-stripping, which is highly operator-dependent as a function of time-expenditure, neuroanatomical knowledge and experience with subsequent intra-/inter-individual variability. Provided that these premises are not given or present a limiting factor, standardised (semi-) automated skull-stripping using ABX can present a beneficial alternative. While our proposed approach is not entirely operator-independent, the results are substantially less subject to intra-/inter-rater variability compared to manual skull-stripping (as shown in Fig. 4).

We evaluated our proposed method in comparison to several other technical approaches including Marker based watershed scalper (MBWSS), Brain-Extraction-Tool v2 (BET), 3dSkullStrip (3DSS), Primatologist-Toolbox (PRIMA), Rapid Automatic Tissue Segmentation (RATS) and Robust automatic rodent brain extraction using 3D pulse-coupled neural networks (PCNN3D). In certain datasets, some of the alternative technical methods were able to provide accurate results, however, failed in other conditions, which is, inter alia, attributable to variable field inhomogeneity and differences in contrast pattern^1^. Unlike other technical implementations using convoluted inferences derived from intensity, morphology or surface, template-based methods like ABX present a generic approach with greater versatility regarding interspecies differences in brain size, shape and tissue contrast. The current study demonstrated that our proposed approach shows robust performance regardless of difference in the degree of sulcus folding (macaque vs. marmoset), brain size (marmoset vs. rodent), developmental time course (juvenile vs. adult marmosets) and contrast pattern (T1/T2 or 3 T/7 T/9.4 T). Moreover, template-based approaches allow for simple implementation of specific segmentation protocols with in- or exclusion of distinct brain regions, such as the brain stem, optic chiasm or cerebellum.

But these benefits come at the cost of high computational demand (typically a few hours) and the time investment of a single manual operation, which is still considerably less manual effort than manual (hybrid) skull-stripping of an entire dataset. In contrast, computational workload and computation time was for the herein tested alternatives much lower (a few minutes). Although, this downside becomes debatable with regard to the likelihood for laborious manual intervention, the growing availability of advanced computing systems in research environments and faster non-linear registration algorithms using multithreading and GPU computing. With regard to the importance of skull-stripping for further processing and data analysis, it is common that reasonable amount of effort is dedicated towards achieving accurate results. However, where dedicated technical approaches, such as PCNN3D for rodents, achieve accurate and robust results within a fraction of computation time, suchlike methods should be considered. In the near future an emerging number of methods will leverage the potential of artificial intelligence (AI), e.g. using machine learning (ML) algorithms and convolutional neural networks (CNN), however, it remains controversial whether these methods will be able to provide versatile performance across MRI protocols, species and populations.

Study-specific templates provide the advantage of accounting for inter-subject variation and providing favourable contrast pattern, which can improve rigid and non-rigid deformation. A hierarchical group-wise registration framework, Atlas Building by Self-Organized Registration and Bundling (ABSORB)^14^, was utilised for unbiased anatomical template building: ABSORB provides accurate templates with sharp structural boundaries using an iterative hierarchical group-wise registration approach, where images are warped towards a set of eligible neighbours and representative images from clusters advance to higher levels. In opposition to other template-based methods, which require either multiple atlases or laborious preparation of probabilistic maps, ABX achieves simple and straightforward skull-stripping with unbiased anatomical atlas building from the study-cohort.

In conclusion, we showed that ABX facilitates robust skull-stripping across T1-/T2-weighted datasets from different species (macaques, marmosets and rodents) at different developmental stages. Due to its generic nature, our proposed approach should be foremost considered for animal MRI studies, where no superior or equivalent dedicated technical method is available. Moreover, it is suitable for neurodevelopmental studies considering the vast morphological changes throughout brain development^18,19^, which can be challenging for brain extraction algorithms. Due to implementation as a scriptable (semi-) automated processing pipeline, large-scale, high-throughput application in functional and structural MRI studies is feasible. While ABX makes use of existing neuroimaging frameworks, the provided solution is not implemented in the respective applications and is novel among the technical methods applied. In addition, implementation using popular neuroimaging frameworks may benefit from their ongoing development, such as improvements regarding registration algorithms or parallel computing. Limitations of the current study are (to some extent) small sample size and evaluation restricted to non-human primates and rodents. Future studies should include larger sample size, in particular from open animal neuroimaging databases, which only started gaining in popularity in recent years^15,20^.

## Data Availability

Authors confirm that all relevant data are included in the article.
